# Integrating Irreversible Thermodynamics and Response Surface Methodology to Elucidate Nitrate Transport in Nanofiltration and Reverse Osmosis Membranes

**DOI:** 10.3390/membranes16030090

**Published:** 2026-03-02

**Authors:** Hajar Zeggar, Soufian El-Ghzizel, Mustapha Tahaikt, Mohamed Taky

**Affiliations:** Laboratory of Advanced Materials and Process Engineering, Faculty of Sciences, Ibn Tofail University, Kenitra P.O. Box 1246, Morocco; soufian.el-ghzizel@uit.ac.ma (S.E.-G.); mustapha.tahaikt@uit.ac.ma (M.T.)

**Keywords:** nitrate, convection and diffusion, Kedem–Katchalsky model, Spiegler–Kedem model, RSM

## Abstract

This study employs an integrated modeling approach to elucidate the mechanisms of nitrate ion transport through nanofiltration (NF) and reverse osmosis (RO) membranes. The investigation first applied models from irreversible thermodynamics, specifically the Kedem–Katchalsky and Spiegler–Kedem models, to describe convective/diffusive contributions and the impact of the initial nitrate concentration (50–150 mg/L) on phenomenological parameters (reflection coefficient σ, and solute permeability P_s_). The results revealed a marked sensitivity of NF membranes to the initial nitrate concentration, in contrast to the stable performance of RO membranes. To deepen this analysis, Response Surface Methodology (RSM) was used as a robust statistical tool to systematically model and quantify the synergistic effects of the initial concentration and other key operational parameters, transmembrane pressure (TMP) and recovery rate (Y) on NF performance. The results highlight the complementarity between transport modelling and statistical approaches for analysing nitrate rejection and permeate flux. The proposed approach provides useful insight into NF membrane-specific behaviour and relative sensitivity to operating conditions, within the scope and limitations of the studied membrane and experimental configurations.

## 1. Introduction

Despite the relatively stable global volume of freshwater, demographic growth, climate change, and industrial pollution continue to intensify pressure on available water resources. According to a joint UNICEF and World Health Organization (WHO) report, nearly 2.2 billion people still lack access to safe drinking water, while more than 780 million, about 10% of the global population, rely on unsafe or unimproved water sources [1,2]. Groundwater, particularly in semi-arid and arid regions, represents a vital supply source but is increasingly threatened by nitrate contamination. This issue has been reported in more than 290 regions worldwide, with approximately 60 identified as critical hotspots, including areas in Brazil, Morocco, Palestine, Pakistan, India, and China [3,4,5,6,7,8].

Most nitrate pollution originates from anthropogenic sources such as excessive fertilizer use, livestock waste, industrial effluents, and landfill leachates [9,10]. Elevated nitrate levels pose significant risks: environmentally, they promote to eutrophication, algal blooms, and hypoxic conditions [11]; for human health, they are linked to methemoglobinemia in infants, spontaneous abortions, thyroid disorders, diabetes, and increased risks of gastric cancer [12]. The sharp rise in nitrate-related publications, from about 1000 in 2002 to over 18,000 in 2024 (ScienceDirect), underscores the growing global concern over this contaminant [13].

To address this challenge, numerous treatment techniques have been investigated. Among them, pressure-driven membrane processes, particularly nanofiltration (NF) and reverse osmosis (RO), are well-established membrane technologies that continue to be extensively applied for nitrate removal [14,15,16]. Reverse osmosis can achieve near-complete nitrate removal; however, its application is often constrained by high operating pressures, energy consumption, and associated costs [17]. Recent advances in membrane science have focused on the development of chemically robust and pH-resistant nanofiltration membranes capable of operating under harsh chemical environments. Acid–alkali tolerant NF membranes have been reported to exhibit enhanced stability while maintaining competitive selectivity and permeability, thereby expanding the operational window of NF processes [18]. In this context, a deeper understanding of ion transport mechanisms, especially for regulated species such as nitrate, remains essential to fully exploit the potential of these advanced membrane materials. The performance of NF and RO systems is governed not only by operating conditions (transmembrane pressure, feed concentration, and recovery rate), but also by intrinsic membrane properties such as molecular weight cut-off (MWCO), selective layer density, and surface charge) and feedwater composition (ionic strength, ion type, and concentration).

Beyond experimental studies, modeling approaches play a crucial role in advancing mechanistic understanding and predictive capability of solute transport through NF and RO membranes. Several theoretical frameworks have been developed, including irreversible thermodynamics, solution–diffusion, steric hindrance, and extended Nernst–Planck models [19,20,21,22,23]. Among these, the Kedem–Katchalsky (KK) and Spiegler–Kedem (SK) models, derived from nonequilibrium thermodynamics, are widely applied to describe the coupled transport of solvent and solute across semi-permeable membranes.

These models are grounded in the principles of irreversible thermodynamics, where mass transfer is governed by the combined effects of diffusion (solute motion under concentration gradients) and convection (flow induced by pressure gradients). The KK model introduces phenomenological coefficients linking measurable parameters such as solvent flux and permeate concentration to the driving forces [24,25]. The SK model further refines this approach through two characteristic parameters: the reflection coefficient (σ) and the solute permeability (P_s_) [26,27]. These models provide physically meaningful transport parameters and have proven useful for interpreting membrane behaviour under controlled experimental conditions. However, they are most often applied independently of systematic optimization methodologies and are typically limited to descriptive or predictive analyses.

In parallel, statistical tools such as Response Surface Methodology (RSM) have gained prominence for optimizing membrane processes. RSM is a robust technique that explores the relationships between multiple operating parameters and response variables, allowing optimization with fewer experiments than conventional approaches [28]. Its ability to generate response surfaces and contour plots offers valuable insights into variable interactions and supports the identification of optimal operating windows [29], and it has been effectively applied to optimize parameters such as pressure, concentration, and recovery rate in membrane processes [30,31,32,33]. While RSM is effective for identifying optimal operating windows, it does not provide mechanistic insight into the underlying transport phenomena.

To address this limitation, an analytical framework is proposed in which phenomenological transport modelling and statistical optimization are structurally integrated. The originality of this work lies in the systematic coupling of the KK and SK transport models with RSM, enabling intrinsic membrane transport parameters to be directly linked to process-level optimization. This integrated strategy goes beyond the conventional use of either transport modelling or statistical optimization alone and provides complementary and deeper insights into membrane performance that cannot be achieved when these approaches are applied separately.

In this work, nitrate transport through a commercial NF membrane (NF90) and a RO membrane (BW30) is investigated. Transport parameters are first determined under controlled conditions using KK and SK models. Subsequently, RSM is applied to evaluate and optimise the performance of the NF membrane under recirculation mode, representative of practical operating conditions. The comparative analysis highlights membrane-specific behaviour, parameter sensitivity to operating conditions, and the respective applicability of NF and RO for nitrate removal. The conclusions drawn are restricted to the studied membranes and experimental configurations.

## 2. Materials and Methods

### 2.1. Characteristics of the Feed Water

#### 2.1.1. Real Water

The water used in this study was slightly brackish groundwater collected from Bejaâd (Khouribga province, Beni Mellal–Khenifra region). According to the analytical results presented in Table 1, this water exceeds the drinking-water quality standards due to its high nitrate concentration (>50 mg/L).

#### 2.1.2. Synthetic Feed Water for Nitrate Transport Study

The synthetic feed solutions were prepared by dissolving sodium nitrate (NaNO_3_) in distilled water to obtain nitrate concentrations of 50, 75, 100, and 150 mg/L. The physicochemical characteristics of these solutions are presented in Table 2.

### 2.2. Description of the Pilot Unit and Membranes Used

**Figure 1:**
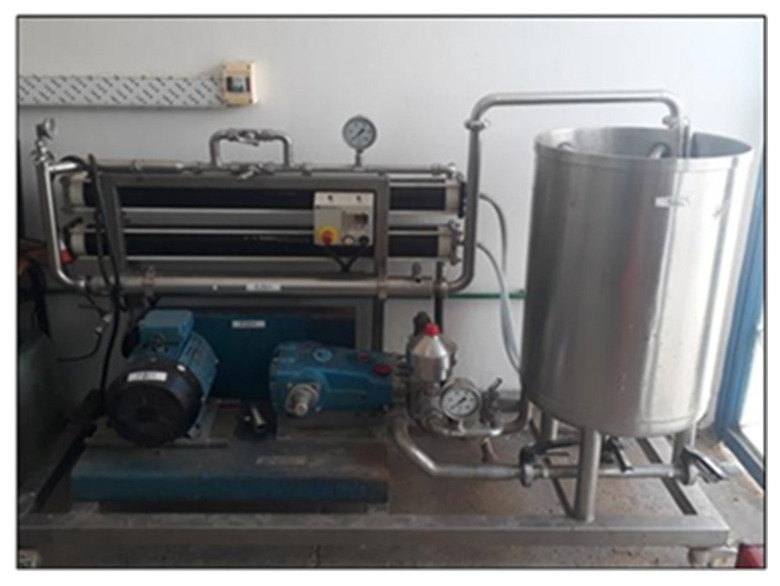
NF/RO pilot.

The experiments were carried out using an NF/RO pilot unit (E 3039) supplied by Applied Industrial Technologies (Bollene, France), as shown in Figure 1. The unit is equipped with two identical pressure vessels connected in series in a rejection configuration, each housing a spiral-wound membrane module. The total pressure drop across the system is approximately 2 bar, corresponding to about 1 bar per vessel. For nanofiltration experiments, both pressure vessels were fitted with identical NF membranes (two NF modules in series). Similarly, for reverse osmosis experiments, both pressure vessels were equipped with identical RO membranes (two RO modules in series). Nanofiltration and reverse osmosis experiments were conducted separately, using the same pilot unit but different membrane sets. During the experiments, three operating parameters were continuously monitored:

▪ Solvent flux (*J_v_*): represents the rate at which water molecules are transported across the membrane per unit time and per unit membrane area.

(1) $$J_{v}=\frac{Q_{p}}{A∆t}$$

▪ Recovery rate (*Y*): corresponds to the fraction of feed water converted into permeate (treated water).

(2) $$Y=\frac{Q_{p}}{Q_{f}}$$

▪ Rejection represents the membrane’s ability to retain solutes and prevent them from passing into the permeate. For each solute *i* that can be retained by the membrane, the rejection coefficient is defined as:

(3) $$R_{i}=1-\frac{C_{pi}}{C_{fi}}$$

where *C_fi_* and *C_pi_* denote the feed and permeate concentrations of component *i*, respectively.

The membranes used in this work are commercial spiral-wound modules manufactured by DOW Chemical (Midland, MI, USA). Their main technical specifications are summarized in Table 3.

### 2.3. Filtration System Configuration

#### 2.3.1. Configuration Using the Irreversible Thermodynamics Approach

**Figure 2:**
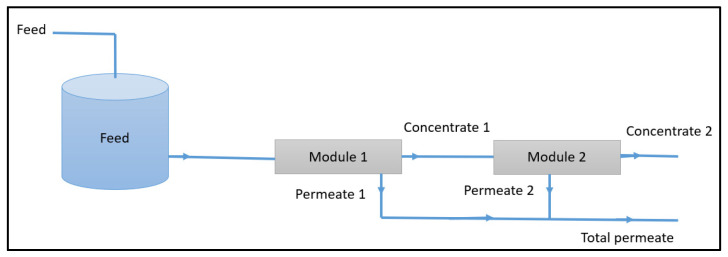
Schematic of the adopted configuration using the irreversible thermodynamics approach.

For the application of the irreversible thermodynamics models, the filtration system was operated without retentate recirculation in order to ensure simple and well-controlled experimental conditions. The feed solution was pumped from the reservoir into the first module, and the concentrate stream was subsequently directed to the second module. The permeates from both modules were collected and combined, as illustrated in Figure 2.

This configuration was selected to maintain an almost constant feed concentration during the experiments, thereby avoiding solute accumulation and time-dependent variations in the driving forces. Such conditions are required for the reliable estimation of intrinsic membrane transport parameters. Membrane cleaning was performed when a decline in demineralization performance was detected based on periodic measurements of pure water permeability. The measured permeability was compared with the manufacturer’s reference value, and deviations were used as an indicator of fouling. The cleaning protocol consisted of alternating acidic and alkaline cleaning steps, each followed by thorough rinsing.

#### 2.3.2. Configuration Using RSM Approach

**Figure 3:**
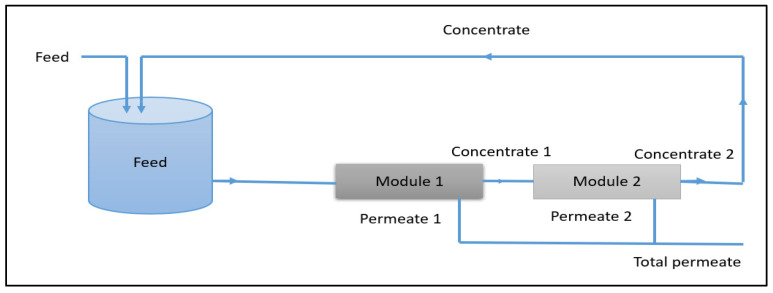
Schematic of the adopted configuration using RSM approach.

For the response surface methodology, the treatment unit (TIA) was operated in semi-batch mode with retentate recirculation, which is more representative of practical membrane operation. In this configuration, the permeate was continuously collected, while the retentate was recycled back into the feed tank, as shown in Figure 3.

This configuration was adopted to evaluate process performance under quasi-steady conditions and to analyse the influence of key operating variables, namely transmembrane pressure (TMP), feed concentration, and recovery rate, as well as their interactions. The use of recirculation enables a realistic assessment of global performance indicators such as permeate flux and nitrate rejection and provides experimental data suitable for statistical optimization.

### 2.4. Analytical Methods

The EC and ionic composition of feed water samples were determined using standardized analytical methods, summarized in Table 4.

## 3. Description of the Models

### 3.1. Modeling Approach Based on Irreversible Thermodynamics (KK and SK Models)

#### 3.1.1. Kedem–Katchalsky Model

In the late 1950s, Kedem and Katchalsky [27] developed a practical transport model based on the linear framework of irreversible thermodynamics. Initially developed to describe passive transport across biological membranes, this model has since been widely applied to pressure-driven membrane processes involving simultaneous solvent and solute transfer.

(4) $$J_{diff}+J_{v}C_{conv}=C_{p}J_{v}$$

Equation (4) can be expressed as follows:

(5) $$C_{p}=\frac{J_{diff}}{J_{v}}+C_{conv}$$

where *J_v_*: solvent flux (L.m^−2^.h^−1^), *C_p_*: permeate concentration (mg/L), *J_diff_*: diffusive flux (mg.m^−2^.h^−1^), *C_conv_*: convective concentration (mg/L).

The KK model provides a global description of transport across the membrane, treating transport parameters as lumped quantities. It does not explicitly account for solute concentration gradients within the membrane thickness. Consequently, it is particularly suitable for simplified analysis and for estimating intrinsic transport parameters under controlled experimental configurations

#### 3.1.2. Spiegler–Kedem Model

The Spiegler–Kedem model, developed by J. Spiegler and O. Kedem [26] in the 1960s as an extension of the original KK formalism, provides a more detailed description of coupled solute and solvent transport through membranes. Unlike the KK model, which is based on global transport relationships, the SK approach relies on local transport equations applied across the membrane thickness, allowing internal concentration gradients to be explicitly taken into account.

Within this framework, the membrane is characterized by phenomenological parameters such as the reflection coefficient (*σ*) and the solute permeability (*P_s_*). The local fluxes, whether of the solvent (*J_v_*) or of a solute dissolved in the solvent (*J_s_*), can be expressed independently according to the following equations:

(6) $$J_{v}=\;-L_{p\;}\left(\frac{dp}{dx}-\sigma \frac{d\pi }{dx}\right)$$

(7) $$J_{s}={-P}_{local}\frac{dC}{dx}+\left(1-\sigma \right)J_{v}C$$

where *Lp*: membrane permeability to solvent, *P*: pressure, *x*: coordinate along the membrane thickness axis. *C*: solute concentration *π*: osmotic pressure due to the solute *P_local_*: the local permeability of the solute.

Assuming that *P_local_* and *σ* are independent of the concentration and that *P_s_* = *P_local_*/Δ*x*, integration of Equations (6) and (7) was achieved with the following limiting conditions: *C* = *C_m_* when *x* = 0 and *C* = *C_p_* when *x* = Δ*x* (the membrane active layer thickness). Then the real rejection of the solute can be calculated by the following equation:

(8) $$R_{real}=1-\frac{1-\sigma }{1-\sigma \;exp\left\lfloor \left(\sigma -1\right)\left(\frac{J_{v}}{P_{s}}\right)\right\rfloor }$$

### 3.2. Statistical Modeling Using RSM

RSM is an empirical statistical approach for process optimization, enabling the systematic assessment of multiple variables and their interactions through a reduced number of experiments.

The relationship between input variables (*x_ᵢ_*) and response (*Y*) is described by a second-order polynomial model:

(9) $$Y=a_{0}+\sum_{i}^{k}a_{i}x_{i}+\sum_{i}^{k}a_{ii}x_{i}^{2}\sum_{i.j=1.j\neq i}^{k}a_{ij}x_{i}x_{j}$$

where *Y* is the response variable, *x_ᵢ_* and *x_ⱼ_* are coded independent variables, and a_0_, a_ᵢ_, a_ᵢᵢ_, a_ᵢⱼ_ are regression coefficients [45].

In this study, the NF process was evaluated using RSM with a Central Composite Design (CCD) to gain a deeper understanding of the transport phenomena involved and to provide a practical framework for optimizing nanofiltration performance in nitrate removal.

Three key factors were studied: feed nitrate concentration [NO_3_^−^]_int_ transmembrane pressure TMP, and recovery rate Y at two coded levels (−1 and +1), as shown in Table 5.

Two response variables were evaluated: permeate flux and nitrate rejection. The experimental design, generated using Design Expert software version 13, consisted of 20 experimental runs, ensuring a comprehensive exploration of the operating domain.

## 4. Result and Discussion

### 4.1. Nanofiltration vs. Reverse Osmosis for Nitrate Removal from Slightly Brackish Groundwater

**Figure 4:**
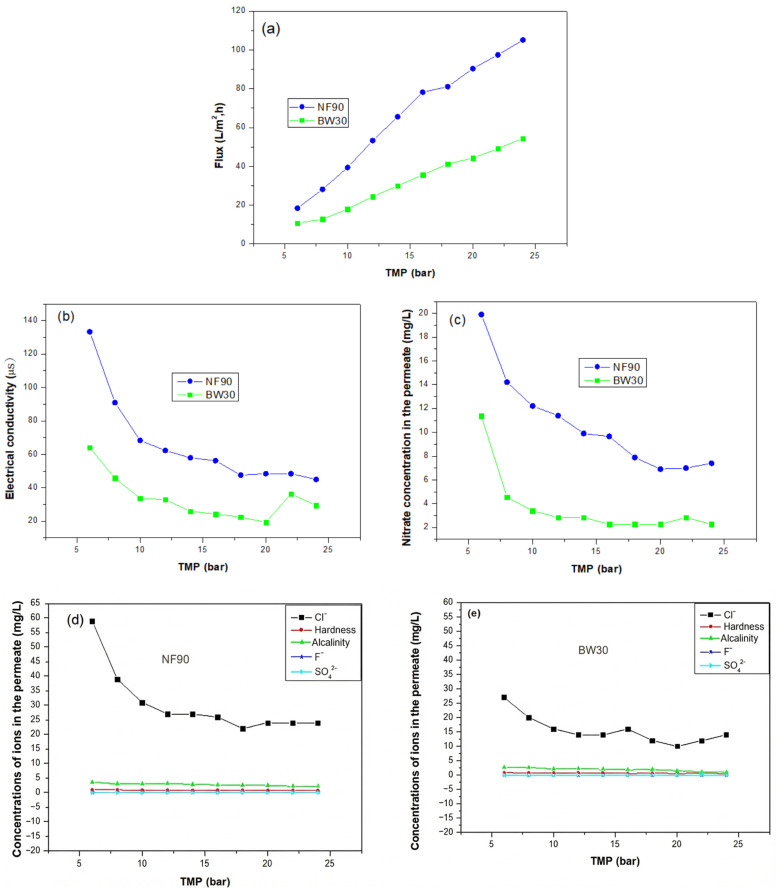
Permeate characteristics as a function of transmembrane pressure (TMP). (**a**) Variation in permeate flux with TMP for NF90 and BW30 membranes, (**b**) Evolution of permeate electrical conductivity as a function of TMP, (**c**) Nitrate concentration in the permeate as a function of TMP, (**d**) Permeate ion concentrations obtained with the NF90 membrane as a function of TMP, (**e**) Permeate ion concentrations obtained with the BW30 membrane as a function of TMP.

Figure 4 illustrates the evolution of permeate flux, permeate conductivity, nitrate concentration, and the concentrations of other ions as a function of applied pressure for the two membranes.

The experimental flux shows a near-linear dependence on pressure for both membranes (Figure 4a), indicating good agreement with Darcy’s law.

In terms of permeate quality, a classic trade-off between permeability and selectivity was observed. The permeate conductivity, used as an indicator of the total dissolved solids, decreased with increasing TMP for both membranes (Figure 4b). This trend is consistent with the dilution effect caused by the enhanced solvent flux at higher pressures, which reduces the relative passage of solutes. Nevertheless, the BW30 membrane consistently produced permeate with much lower conductivity across the entire pressure range, highlighting its superior salt rejection capability, an inherent feature of reverse osmosis membranes.

Regarding the target contaminant, nitrate concentration in the permeate decreased significantly with increasing pressure for both membranes (Figure 4c). This behavior is in good agreement with the solution–diffusion and finely porous models, where higher water flux (*J_v_*) limits the convective transport of solutes, resulting in higher apparent rejection. The BW30 membrane achieved nearly complete nitrate removal (>98%), maintaining permeate concentrations well below the Moroccan drinking water standard (50 mg/L). In contrast, the NF90 membrane, although effective, showed a higher degree of nitrate permeation, with rejection values stabilizing around 94%. This difference is mainly due to the tighter molecular sieving and the absence of significant pores in the dense active layer of the RO membrane.

Furthermore, Figure 4d,e reveal noticeable differences in the permeate chloride ion concentrations between the two membranes, while the concentrations of the other ions remain nearly identical. This observation highlights the distinct separation characteristics of NF90 and reverse osmosis BW30 membranes. In the case of NF90, the higher chloride concentration in the permeate reflects its lower rejection efficiency toward monovalent ions, which is mainly due to its relatively larger pore size and partial charge-based selectivity. Conversely, the BW30 membrane exhibits a much lower chloride content in the permeate, confirming its tighter structure and higher electrostatic repulsion capacity, typical of reverse osmosis membranes. The nearly constant and low concentrations of hardness, alkalinity, fluoride, and sulfate ions for both membranes indicate that these species are effectively rejected, regardless of TMP. Their strong exclusion is primarily governed by Donnan effects and steric hindrance, which restrict the transport of ions with larger hydrated radii and higher valence.

In summary, while the NF90 membrane offers a clear advantage in terms of water productivity, the BW30 membrane delivers superior permeate quality by effectively removing all major ionic species. The following sections will further analyze these separation mechanisms through irreversible thermodynamic modeling and assess the optimization of the NF process using advanced statistical tools.

### 4.2. NF/RO Transport Modeling: Effect of Initial Nitrate Concentration

This section focuses on analyzing nitrate ion transport through nanofiltration and reverse osmosis membranes, emphasizing the influence of initial nitrate concentration. By applying irreversible thermodynamics over a concentration range of 50–150 mg/L, the study aims to elucidate the transport mechanisms and highlight the distinct behaviors of both membrane types.

#### 4.2.1. KK Model

To evaluate the respective contributions of convection and diffusion in nitrate ion transport through NF and RO membranes, Equation (5) of the KK model was applied.

**Figure 5:**
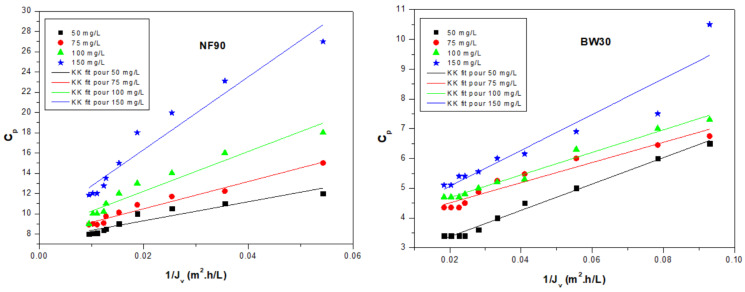
Nitrate ion concentration in the permeate as a function of the inverse of permeate flux for different initial feed concentrations.

Figure 5 illustrates the variation in nitrate concentration in the permeate (*C_p_*) as a function of the inverse of permeate flux (1/*J_v_*). For both membranes, a linear relationship consistent with Equation (5) is observed.

Table 6 reports the values of convective concentration (*C_conv_*), diffusive flux (*J_diff_*), and the coefficient of determination (R^2^) for both NF90 and BW30 membranes at different initial nitrate concentrations (50–150 mg/L).

For the NF90 membrane, both *C_conv_* and *J_diff_* increase markedly with increasing initial nitrate concentration. *C_conv_* rises from 7.41 to 9.13 mg/L, while *J_diff_* increases sharply from 94.14 to 359.49 mg·m^−2^·h^−1^, indicating that diffusion becomes increasingly dominant at higher feed concentrations. The relatively high R^2^ values (0.88–0.93) confirm the strong linearity predicted by the Kedem–Katchalsky model, validating its applicability to describe nitrate transport for NF90.

In contrast, the BW30 membrane exhibits lower *C_conv_* (2.42–3.92 mg/L) and *J_diff_* (33.91–60.32) values across the studied concentration range. Although *C_conv_* increases slightly with initial nitrate concentration, *J_diff_* does not show the same pronounced trend as in NF90, suggesting that the BW30 membrane maintains a more stable diffusive contribution regardless of feed concentration. The R^2^ values (0.88–0.98) also confirm a good agreement with the model.

Overall, NF90 shows higher convective and diffusive contributions than BW30, which can be attributed to its lower salt rejection and higher hydraulic permeability. For both membranes *J_diff_* predominates over *C_conv_*, confirming that diffusion is the main mechanism controlling nitrate transport. However, the sharper increase in *J_diff_* in NF90 indicates greater sensitivity to feed concentration, whereas BW30 demonstrates a more restrictive transport behavior, consistent with its tighter structure and superior nitrate rejection performance. Similar behavior was observed by Zeggar et al. [21], who reported that diffusion dominates nitrate transport, with NF90 showing higher sensitivity to feed concentration than RO.

Overall, these findings emphasize the combined roles of convection and diffusion in nitrate transport, with diffusion identified as the predominant mechanism for both membranes. Furthermore, the NF90 membrane demonstrates a higher sensitivity to variations in the initial nitrate concentration.

**Figure 6:**
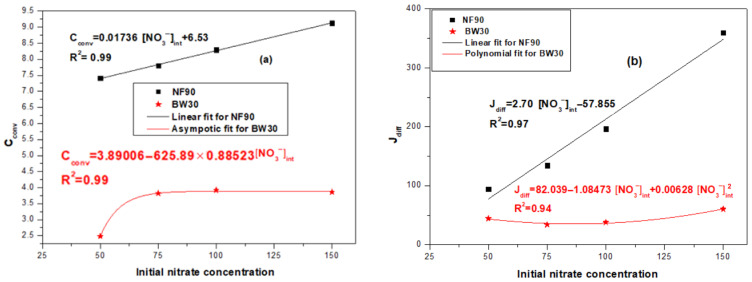
Variation in convective concentration C_conv_ (**a**) and diffusive flux J_diff_ (**b**) as a function of the [NO_3_^−^]_int_.

The impact of the initial nitrate concentration ([NO_3_^−^]_int_) on the convective concentration and diffusive flux through the NF90 and BW30 membranes is investigated and illustrated in Figure 6.

The data in Figure 6a demonstrate that, For NF90, a clear linear relationship (R^2^ = 0.99) is observed, indicating that convective transport increases proportionally with feed concentration. In contrast, BW30 displays an asymptotic response (R^2^ = 0.99), suggesting a near-saturation behavior where further increases in feed concentration do not significantly enhance convective transport. This difference can be interpreted in terms of underlying transport mechanisms. As [NO_3_^−^]_int_ increases, the concentration difference across the membrane becomes more pronounced, thereby enhancing the osmotic pressure gradient. This gradient intensifies the driving force for convective transport, meaning that a greater amount of solute is carried with the solvent flux, thus increasing the convective contribution to the overall transport. This mechanism is consistent with fluid mechanics principles, whereby higher solute concentrations reinforce the coupling between solvent flux and solute transport.

Both NF90 and BW30 are polyamide thin-film composite membranes with comparable surface roughness (72.4 ± 5.8 nm for NF90 and 68.3 ± 12.5 nm for BW30), indicating that surface topography effects are unlikely to account for the observed differences in nitrate transport. Instead, separation behavior is primarily governed by intrinsic membrane properties, notably the molecular weight cut-off MWCO and selective layer density. The looser structure of NF90 (higher MWCO) promotes stronger solute–solvent coupling effects, leading to a higher sensitivity of nitrate rejection to initial nitrate concentration, whereas the denser BW30 membrane (lower MWCO) rapidly reaches a quasi-saturation regime in which nitrate transport becomes less responsive to changes in initial nitrate concentration.

Regarding diffusion, NF90 shows a linear increase in J_diff_ (R^2^ = 0.97), whereas BW30 follows a quadratic (U-shaped) trend, with J_diff_ initially decreasing slightly before increasing again at higher concentrations (Figure 6b).

According to Fick’s law, diffusive flux is proportional to the concentration gradient. As [NO_3_^−^]_int_ rises, the gradient becomes steeper, promoting nitrate migration. In NF90, the less restrictive structure supports continuous diffusion, while in BW30, internal resistance limits diffusion at low concentrations until higher osmotic gradients restore the flux.

Thus, NF90 exhibits linear trends for both C_conv_ and J_diff_, implying enhanced nitrate passage at higher concentrations, whereas BW30 demonstrates nonlinear saturation behavior, limiting nitrate transport even at elevated concentrations. This behavior may result from a shift in the dominant transport mechanism. At low solute concentrations, the rejection is so high that the concentration gradient across the membrane remains minimal, thereby limiting diffusive transport. At higher concentrations, however, the effect of osmotic pressure (Δπ) becomes significant, reducing the net solvent flux and altering the overall transport kinetics. This could explain the observed increase in J_diff_. Such behavior reflects the complex coupling phenomena characteristic of dense membranes.

The deviation from linearity observed for BW30 suggests the influence of factors such as membrane structure, pore size distribution, and stronger electrostatic exclusion mechanisms. These effects align with the solution–friction (SF) theory proposed by Kimani et al. [46], which describes ion transport as governed not only by concentration gradients or pressure but also by frictional and electrostatic interactions within the membrane matrix. These insights highlight the importance of coupled transport effects and membrane-specific properties in explaining deviations from classical linear diffusion models.

#### 4.2.2. SK Model

**Figure 7:**
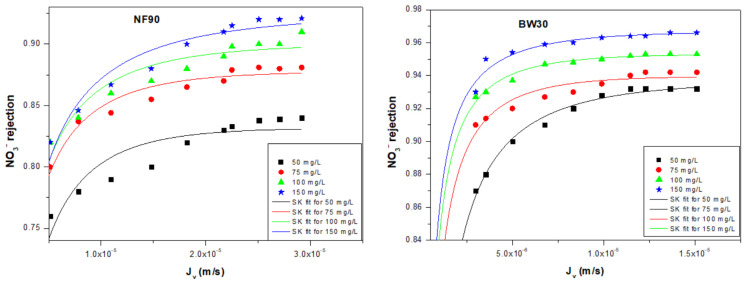
Experimental and fitted data of nitrate rejection as a function of permeate flux for NF90 and BW30 membranes.

Nitrate rejection was evaluated using Equation (8) of the SK model to assess the effect of the initial nitrate concentration on both the contributions of the convective and diffusive transport mechanisms and the phenomenological parameters (σ, P_s_) governing ion transport through the studied membranes. Figure 7 illustrates the variation in nitrate rejection at different [NO_3_^−^]_int_, showing a strong agreement between the experimental measurements and the SK model predictions for both membranes.

The results are summarized in Table 7. For BW30, P_s_ values (on the order of 10^−8^ m·s^−1^) are slightly lower than those of NF90 (10^−7^ m·s^−1^), indicating reduced solute passage through BW30.

The high σ values for both membranes confirm strong nitrate rejection and indicate that diffusive transport dominates over convective transport. These findings are consistent with those obtained from the KK model and align well with literature data [47].

**Figure 8:**
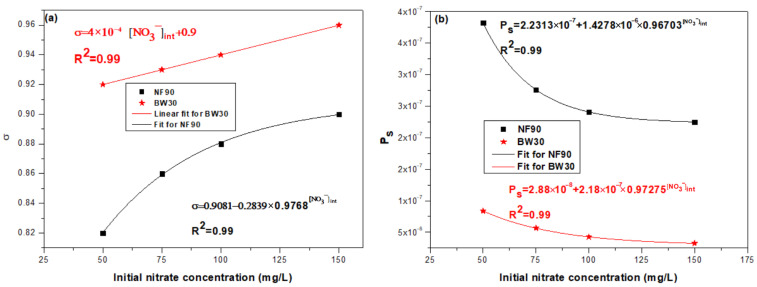
Reflection coefficient σ (**a**) and Nitrate permeability Ps (**b**) as a function of [NO_3_^−^]_int_ for NF90 and BW30 membranes.

The effect of the initial nitrate concentration [NO_3_^−^]_int_ on the reflection coefficient σ and nitrate permeability Pₛ was investigated for the NF90 and BW30 membranes (Figure 8). As shown in Figure 8a, σ increases with [NO_3_^−^]_int_ for both membranes. The increase is approximately linear for BW30, indicating stable and predictable behavior, while NF90 exhibits an asymptotic trend, suggesting that the membrane approaches a limiting selectivity without reaching σ = 1. Regarding nitrate permeability (Figure 8b), both membranes show a decreasing trend with increasing [NO_3_^−^]_int_, following a non-linear relationship.

The observed differences in σ and Pₛ between NF90 and BW30 are related to their intrinsic structural characteristics. NF90 has a higher molecular weight cut-off (MWCO ≈ 200–400 Da) and a looser selective layer, resulting in higher sensitivity to concentration and ionic strength variations. In contrast, the denser BW30 membrane (MWCO ≈ 100 Da) maintains more stable rejection and lower solute permeability.

The increase in [NO_3_^−^]_int_ also raises the ionic strength of the feed solution, compressing the electrical double layer at the membrane surface and modifying electrostatic interactions between the membrane and nitrate ions. This effect contributes to the increase in σ and the decrease in Pₛ, particularly for NF90. Similar concentration-dependent transport behaviors have been reported for NF and RO membranes, confirming that membranes with lower selectivity exhibit stronger variations in transport parameters as a function of solute concentration and ionic strength [48,49].

In summary, the phenomenological parameters of the NF90 and BW30 membranes are strongly dependent on the initial nitrate concentration. NF90 displays a more pronounced and asymptotic response, indicating its higher sensitivity to concentration variations, whereas BW30 shows a more regular and quasi-linear response, reflecting its superior nitrate rejection capability.

### 4.3. Modeling of Nanofiltration Performance by RSM

The previous section showed that nanofiltration performance is highly sensitive to the initial nitrate concentration. In this section, Response Surface Methodology (RSM) is applied to gain deeper insights into the performance of nanofiltration membranes and their dependence on the initial nitrate concentration. The results obtained with the NF membrane are summarized in Table 8, which provides valuable information on process behavior and enables the identification of the most significant parameters and their interactions.

#### 4.3.1. Effect of Operating Parameters on Permeate Flux

Table 9 presents the ANOVA results for the permeate flux response. The model exhibits a high F-value coupled with an extremely low *p*-value, indicating that it is statistically significant and that the variability explained by the model is unlikely to have occurred by chance. The coefficient of determination (R^2^) is remarkably high, demonstrating that the model successfully captures nearly all of the observed variability in the flux data. Furthermore, the high adjusted R^2^ value confirms the robustness of the model, suggesting that it provides a reliable fit without evidence of overfitting. Together, these statistical indicators validate the suitability of the regression model for describing the influence of the studied factors on permeate flux and for guiding process optimization.

The model coefficient estimates and their significance are presented in Table 10. According to the statistical analysis, terms with *p*-values below 0.005 are considered significant, whereas higher values indicate non-significant effects [50,51].

The regression equation for the permeate flux of the NF membrane expressed in terms of actual variables as shown in Equation (10).

(10) $$\begin{array}{ll} Flux=3.8757 & -\;0.062362\;{\left[{{NO}_{3}}^{-}\right]}_{int}+4.42581\;PTM\\ & +\;0.206747\;{\left[{{NO}_{3}}^{-}\right]}_{int}\times Y \end{array}$$

The model coefficients provide further insight: the intercept (88.92) represents the baseline flux when all coded factors are at their central levels (0). The coefficient for [NO_3_^−^]_int_ (−0.062362) indicates a slight negative effect of increasing [NO_3_^−^]_int_ on the flux. The coefficient for TMP (+4.42581) is strongly positive, showing that the TMP is the most influential factor on flux. The coefficient for the interaction [NO_3_^−^]_int_ *Y (+0.206747) confirms that the combined effect of [NO_3_^−^]_int_ and Y is positive, even though Y alone is not significant. Overall, these findings indicate that TMP predominantly controls the permeate flux in nanofiltration, which is consistent with the typical behavior of pressure-driven membrane processes. The negative effect of [NO_3_^−^]_int_ and the positive effects of TMP and the [NO_3_^−^]_int_ × Y interaction emphasize the importance of optimizing both individual factors and their interactions to maximize nitrate removal.

**Figure 9:**
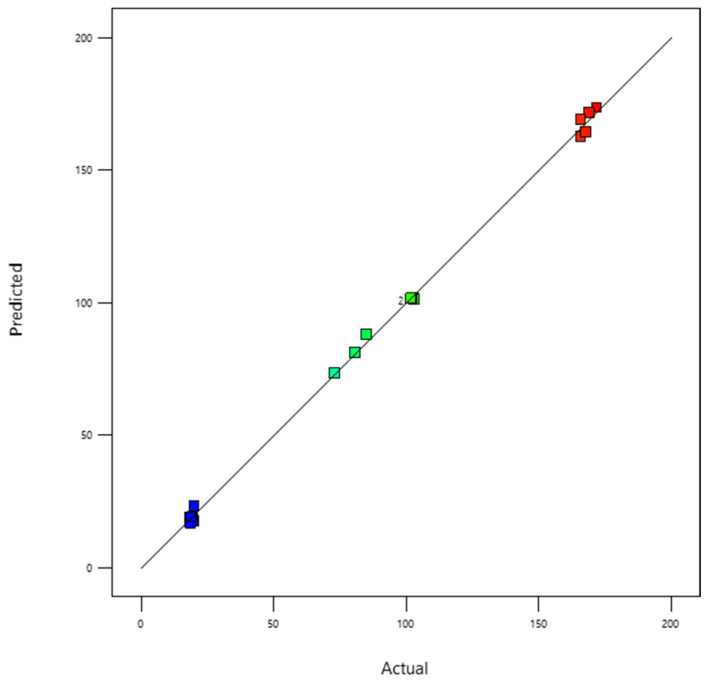
Predicted versus experimental values for the response “permeate flux”. The colored squares represent different experimental runs corresponding to the levels of the RSM design. The color scale ranges from blue (lower values) to red (higher values), which is a standard representation in response surface methodology to visualize the variation of the response.

Figure 9 illustrates the plot of predicted versus experimental values for the permeate flux. The data points closely follow the diagonal line, indicating a strong agreement between the model predictions and the experimental measurements across the entire studied range. Residual analysis further confirms the model’s adequacy: the residuals are randomly distributed around zero, with no apparent patterns or systematic deviations, suggesting that the errors are purely random. This indicates that the model reliably captures the behavior of the nanofiltration process and can be confidently used for process optimization and prediction of permeate flux under varying operating conditions.

**Figure 10:**
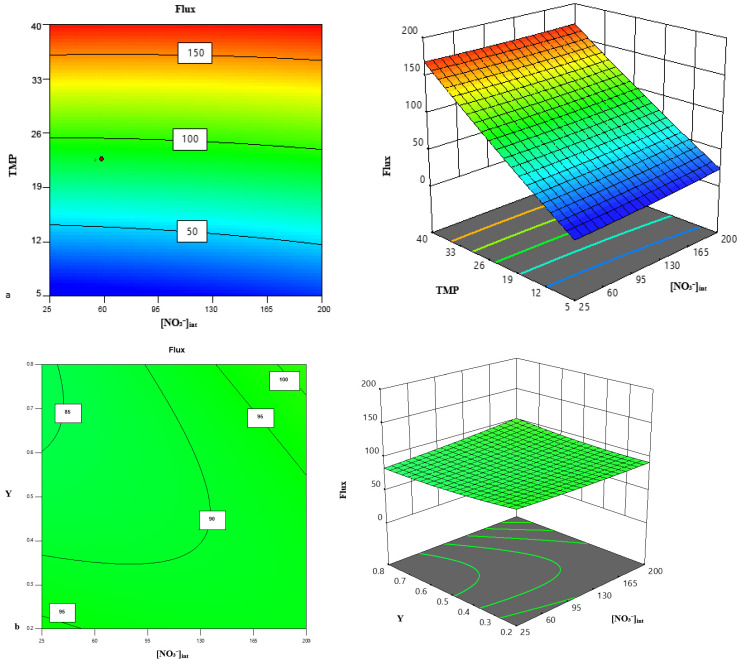

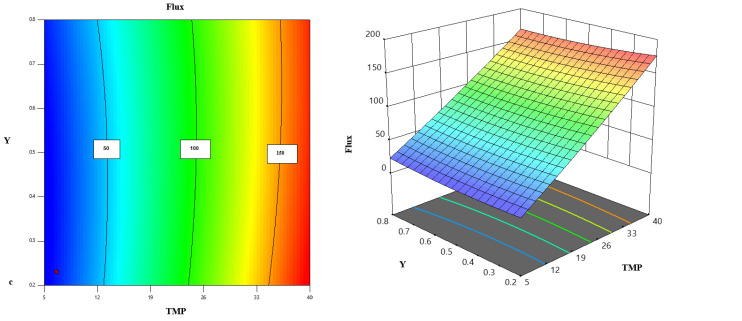
Response surface plots and contour lines of the flux as a function of PTM and [NO_3_^−^]_int_ (**a**), [NO_3_^−^]_int_ and Y (**b**), and PTM and Y (**c**). The color scale ranges from blue (lower values) to red (higher values), which is a standard representation in response surface methodology to visualize the variation of the response.

To investigate the effects of the operating parameters and their interactions on the permeate flux of the NF membrane, both three-dimensional (3D) response surface plots and two-dimensional (2D) contour plots were employed to illustrate the combined effects of the three parameters [NO_3_^−^]_int_, TMP and Y on permeate flux (Figure 10). The 3D response surfaces provide a clear visual representation of how pairs of factors jointly influence flux, while the contour plots offer a simplified 2D perspective for easier interpretation. To facilitate visualization and interpretation, one of the three variables was held constant at its central level while the remaining two variables were varied across their studied ranges. This method allows for detailed visualization of the interactions between parameters and their influence on process performance.

As shown in Figure 10a,c, an increase in TMP exerts a positive effect on the permeate flux. This trend is consistently observed under all studied conditions, confirming that TMP is the key factor governing flux [52]. This observation is consistent with Darcy’s law for membrane filtration, which states that flux is directly proportional to TMP [53]. Based on the response surface analysis, the effect of the interaction term [NO_3_^−^]_int_ *Y was found to have only a minor influence on the permeate flux, consistent with the trend identified by the regression model (Figure 10b). Although the regression model detected a slight effect of [NO_3_^−^]_int_, the response surface plots (Figure 10c) did not reveal any substantial impact of this parameter on flux. This may be because the studied concentration range was not wide enough to produce a strong osmotic gradient or significant concentration polarization effects. As a result, variations in [NO_3_^−^]_int_ do not cause a notable change in flux within the tested domain.

Furthermore, the dominant contribution of TMP as the main driving force likely overshadowed the weaker influence of solute concentration. Hence, TMP is confirmed as the key governing parameter, while [NO_3_^−^]_int_ × Y plays only a secondary role.

#### 4.3.2. Effect of Operating Parameters on Nitrate Rejection

Table 11 and Table 12 present the results of the ANOVA, which allow for the assessment of the significance of both the individual factors and the overall model. The F-value and the corresponding *p*-values were employed to evaluate statistical significance. The model was found to be highly significant (*p*-value < 0.0001). Among the individual factors, only A and C exhibited statistical significance, in addition to the quadratic term associated with factor A, which was also included in the model. Furthermore, the coefficient of determination (R^2^) indicates that the model accounts for a substantial proportion of the observed variance.

The regression equation for the nitrate rejection of the NF membrane expressed in terms of actual variables as shown in Equation (11).

(11) $$\begin{array}{cc} Nitrate\;rejection & \\ = & -0.177434+0.011343{\left[{{NO}_{3}}^{-}\right]}_{int}+1.03834Y\\ - & 0.000034{{\left[{{NO}_{3}}^{-}\right]}_{int}}^{2} \end{array}$$

The regression equation highlights the combined influence of [NO_3_^−^]_int_ and Y on nitrate rejection. Coefficient analysis indicating that the recovery rate exerts a dominant positive linear effect on the response. In parallel, nitrate concentration exhibits a dual influence: linear at low concentrations and nonlinear at higher levels due to the presence of the quadratic term [NO_3_^−^]_int_^2^. Accordingly, an initial increase in [NO_3_^−^]_int_ enhances nitrate rejection, but beyond a critical threshold, the negative quadratic term leads to a decline in performance. This behavior reflects the existence of an operational optimum, beyond which separation efficiency is limited, most likely due to the combined effects of increased ionic strength and the reduction in the Donnan potential. These results are consistent with trends reported in the literature: several studies have shown that increasing nitrate concentration initially promotes rejection through charge effects, whereas higher concentrations reduce efficiency due to electrical double-layer compression and progressive saturation of the membrane’s active sites [54,55,56,57,58]. Likewise, it is well established that the recovery rate is a critical parameter in NF membrane performance, with higher recovery rate generally deteriorating permeate quality [59,60]. However, in our study, a high recovery rate instead enhanced nitrate rejection. This particularity can be attributed to the experimental configuration adopted, namely a semi-batch system. In such a setup, the permeate volume is gradually withdrawn, leading to an increased concentration of the feed solution and, consequently, an apparent improvement in rejection. Thus, the positive effect observed is not solely attributable to the intrinsic properties of the membrane, but also to the hydrodynamic conditions inherent to the semi-batch configuration, which intensify the ion-exclusion mechanism.

**Figure 11:**
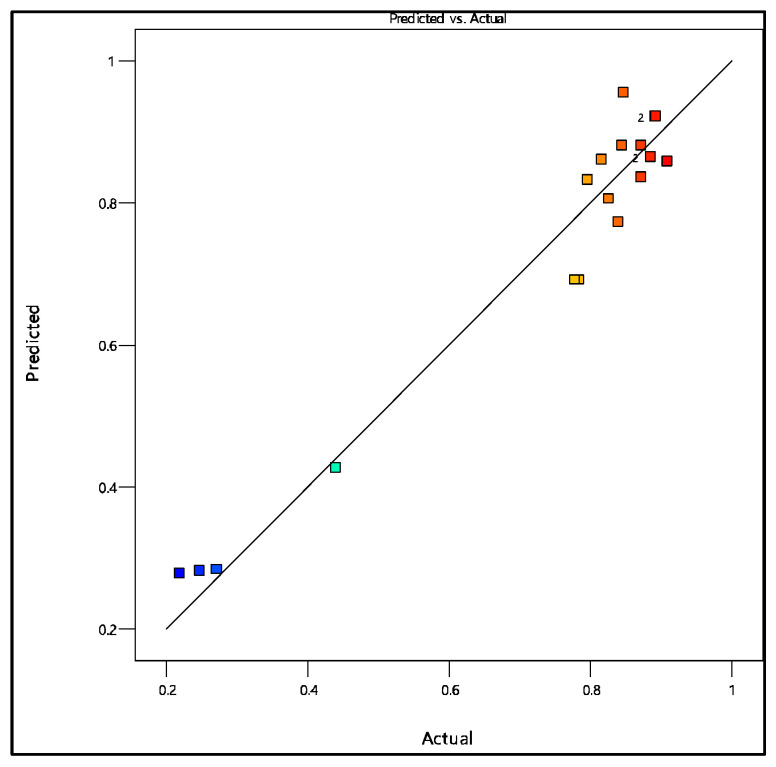
Predicted versus experimental values for nitrate rejection. The colored squares represent different experimental runs corresponding to the levels of the RSM design. The color scale ranges from blue (lower values) to red (higher values), which is a standard representation in response surface methodology to visualize the variation of the response.

Figure 11 illustrates the correlation between the experimental values and those predicted by the regression model. The distribution of data points around the diagonal highlights the strong agreement between observed and calculated values, thereby confirming the predictive capability of the model across the investigated range. The slight deviations observed at higher values remain limited and can be attributed to experimental uncertainties or secondary phenomena not explicitly accounted for in the model. Overall, this graphical representation confirms the robustness and statistical reliability of the developed model, supporting its use for the prediction and optimization of nitrate retention performance.

Overall, this modeling emphasizes the need to simultaneously optimize recovery rate and feed concentration in order to maximize nitrate retention, while complying with the operational constraints of the process.

**Figure 12:**
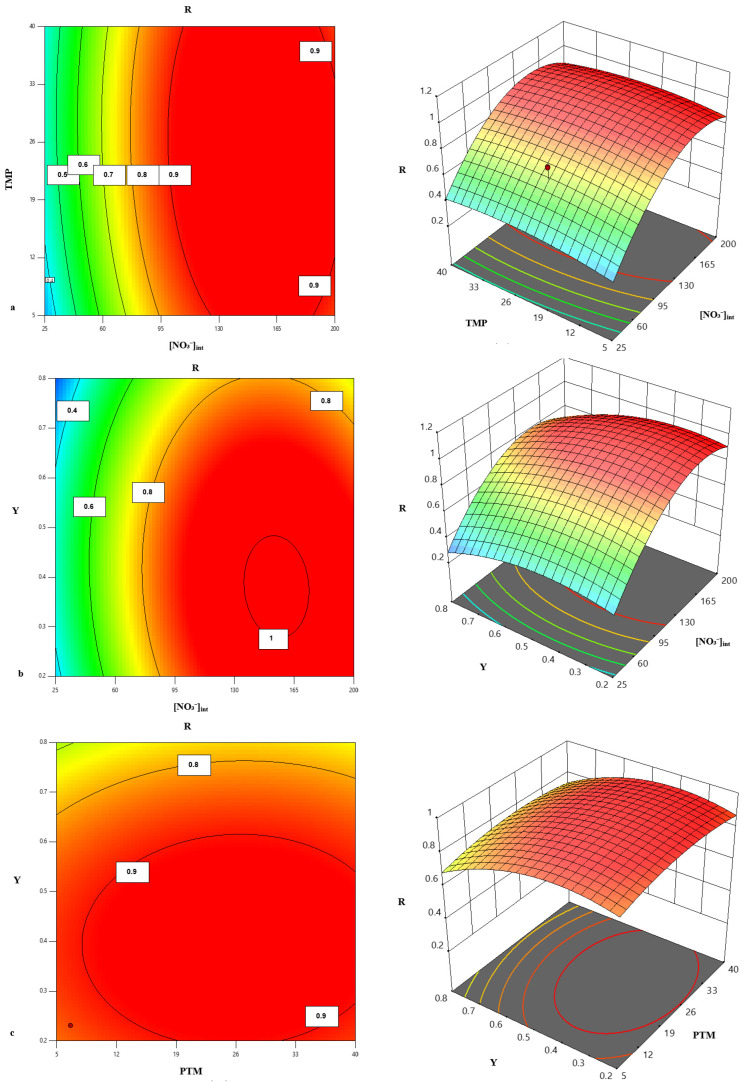
Response surface plots and contour lines of the rejection (R) as a function of PTM and [NO_3_^−^]_int_ (**a**), [NO_3_^−^]_int_ and Y (**b**), and PTM and Y (**c**).

The response surface plots presented in Figure 12a–c illustrates the influence of operating parameters and their interactions on nitrate rejection by NF. As shown in Figure 12a, an increase in [NO_3_^−^]_int_ significantly enhances the nitrate rejection, while TMP also improves nitrate removal. However, beyond 12 bar, the effect of TMP becomes marginal, suggesting that the initial nitrate concentration plays the predominant role. This observation is consistent with previous studies on nanofiltration applied to drinking water treatment [61,62,63].

Figure 12b highlights a significant interaction between [NO_3_^−^]_int_ and Y. The elliptical contour plot identifies an optimal zone where a simultaneous adjustment of these two variables is required to maximize rejection, which corroborates previous findings in the literature regarding the combined influence of operating conditions on nitrate separation by NF [52].

Finally, Figure 12c indicates that a concomitant increase in TMP and recovery rate leads to the highest levels of nitrate rejection.

These trends confirm the results obtained from the analysis of variance (ANOVA) and regression models, demonstrating that nitrate rejection is primarily governed by the initial nitrate concentration and the recovery rate, whereas TMP exerts a more limited effect. These results therefore emphasize the importance of precise control of operating parameters to optimize the efficiency of nanofiltration in nitrate removal.

## 5. Conclusions

This study investigated nitrate transport through nanofiltration (NF90) and reverse osmosis (BW30) membranes by combining phenomenological transport modelling with statistical analysis.

The application of irreversible thermodynamics models, specifically the Kedem–Katchalsky and Spiegler–Kedem formulations, allowed for the detailed deconvolution of coupled transport phenomena, revealing that diffusion is the dominant mechanism governing nitrate permeation. These models quantitatively captured the concentration-dependent selectivity of both membranes, with the NF90 membrane exhibiting markedly higher sensitivity to feed concentration variations compared to the more stable BW30 membrane. This clear contrast highlights the intrinsic trade-off between permeability and selectivity characteristic of pressure-driven membrane processes.

In parallel, the use of Response Surface Methodology (RSM) enabled a systematic optimization of nanofiltration performance, identifying the most influential operational parameters. While the solvent flux was primarily controlled by transmembrane pressure, nitrate rejection was significantly enhanced by both the initial nitrate concentration and, notably, the recovery rate. Moreover, a critical synergistic interaction between concentration and recovery rate was identified, underscoring the importance of multivariate optimization for accurate process control.

Rather than proposing new transport equations, this work demonstrates the complementarity of combining transport modelling with statistical optimization. The thermodynamic models provide mechanistic insight into membrane-specific transport behaviour, while RSM offers a practical framework for evaluating the sensitivity of process performance to operating conditions. The conclusions drawn are restricted to the investigated membranes and experimental configurations.

Future work should extend this integrated approach to multi-ionic feed waters and assess its applicability under long-term operating conditions, including fouling and scaling effects, in order to further support the optimization of NF and RO processes for nitrate removal in practical applications.

## Figures and Tables

**Table 1:** Properties of the Natural Feed Water.

Parameters	Feed Water
Electrical Conductivity (EC) (µs/cm)	1330
NO_3_^−^ (mg/L)	119
Cl^−^ (mg/L)	536
F^−^ (mg/L)	1.2
SO_4_^2−^ (mg/L)	230
TH (F°)	30.66
TAC (F°)	30

**Table 2:** Properties of the Synthetic Feed Water.

**NO_3_^−^ (mg/L)**	50	75	100	150
**EC (µS/cm)**	92.14	137.59	182.08	272.57

**Table 3:** Main characteristics of NF/RO membranes.

Parameter	NF90	BW30
Process	Nanofiltration	Reverse Osmosis
Membrane type	Polyamide; Thin-Film Composite	Polyamide; Thin-Film Composite
Active area (m^2^)	7.6	7.25
Salt passage (%)	5–15% (CaCl_2_)	<3% (MgSO_4_)
Maximum pressure (P_max_)	41 bar	41 bar
Maximum temperature (T_max_)	45 °C	45 °C
pH range (continuous operation)	2–11	2–11
pH range (short-term cleaning, 30 min)	1–12	1–13
Silt Density Index (SDI)	5	5
Free chlorine tolerance	<0.1 ppm	<0.1 ppm
Molecular weight cut-off (MWCO)	200–400	100
Pore size (nm)	0.68 [34]	-
Roughness (nm) [35]	72.4 ± 5.8	68.3 ± 12.5

**Table 4:** Parameters and analytical methods for feed water characterization.

Parameter to Be Measured	Reference Methods
EC	[36]
Salinity	[37]
Fluoride	[38]
Chloride	[39]
Nitrate	[40]
Sulfate	[41]
Hardness	[42]
Alkalinity	[43,44]

**Table 5:** Factors in coded and actual values.

Actual Variables	Unit	Coded Variables	Level (−1)	Level (+1)
[NO_3_^−^]_int_	ppm	A	25	200
TMP	bar	B	5	40
Y	-	C	0.2	0.8

**Table 6:** Values of C_conv_ and J_diff_ obtained at different initial nitrate concentrations in the feed.

Initial Nitrate Concentration (mg/L)		50	75	100	150
NF90	C_conv_ (mg/L)	7.41	7.8	8.29	9.13
	J_diff_	94.14	134.29	196.29	359.49
	R-square	0.88	0.92	0.92	0.93
BW30	C_conv_ (mg/L)	2.42	3.82	3.92	3.86
	J_diff_	44.28	33.91	37.91	60.32
	R-square	0.98	0.96	0.98	0.88

**Table 7:** Values of σ and P_s_ calculated using the SK model for NF90 and BW30 membranes.

Initial Nitrate Concentration (mg/L)		50	75	100	150
NF90	$P_{s}$ $\;(\frac{m^{3}}{m^{2}.s})\times 10^{-7}$	3.82	2.76	2.41	2.25
	σ	0.82	0.86	0.88	0.9
	R-square	0.99	0.99	0.99	0.99
BW30	$P_{s}$ $\;(\frac{m^{3}}{m^{2}.s})\times 10^{-8}$	8.37	5.65	4.25	3.24
	σ	0.92	0.93	0.94	0.96
	R-square	0.99	0.99	0.99	0.99

**Table 8:** Experimental matrix and results.

	Factors			Responses	
Experiments (in Logical Order)	A: [NO_3_^−^]_int_	B: TMP	C: Y	Flux (L/m^2^.h)	Rejection
1	200	40	0.2	172	0.91
2	103.75	5.387	0.5312	19.7	0.79759
3	103.75	24.25	0.209	103	0.872771
4	58.64	22.65	0.5	85.3	0.78513
5	195.625	24.25	0.531	103	0.891629
6	25	5	0.2	18.6	0.272
7	103.75	24.25	0.209	103	0.844819
8	195.62	24.25	0.531	102.4	0.893671
9	25	40	0.8	166	0.248
10	128.25	19.52	0.782	80.8	0.826901
11	195.62	24.25	0.531	101.9	0.893671
12	25	40	0.422	169.3	0.44
13	103.75	5.87	0.5312	19.6	0.872771
14	112.5	6.66	0.23	20	0.816889
15	127.37	38,95	0.449	168	0.847688
16	58.64	22.65	0.5	85	0.778308
17	135.25	40	0.8	166	0.840296
18	25	19.95	0.8	73	0.22
19	200	5	0.2	18.6	0.886
20	200	5	0.2	18.8	0.886

**Table 9:** Analysis of Variance (ANOVA).

Source	Sum of Squares	Degrees of Freedom	Mean Square	F-Value	*p*-Value	Significance
Model	61847.26	9	6871.92	797.79	<0.0001	Significant
Residual	86.14	10	8.61			
Total	61933.4	19				
R^2^	0.9986					
R^2^_ajusté_	0.9974					

**Table 10:** Estimated model coefficients and their significance.

Coefficient	Estimates (Coded Values)	Estimates (Actual Values)	Standard Error	Sum of Squares	F-Value	*p*-Value	Significance
Intercept	88.92	3.8757	1.61	-	-	-	-
A-[NO_3_^−^]_int_	3.74	−0.062362	1.02	115.82	13.45	0.0043	Significant
B-TMP	76.03	4.42581	1.11	40,133.12	4659.22	<0.0001	Significant
C-Y	−0.5892	−48.10365	1.15	2.25	0.2617	0.6201	not Significant
AB	−2.8	−0.001826	1.66	24.57	2.85	0.1221	not Significant
AC	5.43	0.002067	1.96	66.37	7.7	0.0196	Significant
BC	−4.04	−0.768581	1.98	35.8	4.16	0.0688	not Significant
A^2^	1.46	0.00019	1.66	6.64	0.7709	0.4006	not Significant
B^2^	3.46	0.011302	1.65	37.81	4.39	0.0626	not Significant
C^2^	3.62	40.17356	2.01	27.94	3.24	0.1019	not Significant

**Table 11:** Analysis of Variance (ANOVA) for nitrate rejection.

Source	Sum of Squares	Degrees of Freedom	Mean Square	F-Value	*p*-Value	Significance
Model	1	9	0.1113	22.41	<0.0001	Significant
Residual	0.0497	10	0.0050			
Total	1.05	19				
R^2^	0.9528					
R^2^_ajusté_	0.9103					

**Table 12:** Estimated Model coefficients and their significance for nitrate rejection.

Coefficient	Estimates (Coded Values)	Estimates (Actual Values)	Standard Error	Sum of Squares	F-Value	*p*-Value	Significance
Y-intercept	0.94	−0.177434	0.0385	-	-	-	-
A-[NO_3_^−^]_int_	0.2426	0.011343	0.0245	0.4874	98.15	<0.0001	Significant
B-TMP	0.0216	0.009657	0.0267	0.0032	0.6542	0.4374	Not Significant
C-Y	−0.0705	1.03834	0.0277	0.0322	6.49	0.029	Significant
AB	−0.0195	−0.000013	0.0397	0.0012	0.2397	0.635	Not Significant
AC	−0.0284	−0.001081	0.0469	0.0018	0.3651	0.5591	Not Significant
BC	0.0054	0.001035	0.0475	0.0001	0.0131	0.9113	Not Significant
A^2^	−0.2635	−0.000034	0.0398	0.2172	43.73	<0.0001	Significant
B^2^	−0.0511	−0.000167	0.0397	0.0082	1.66	0.2267	Not Significant
C^2^	−0.1057	−1.17492	0.0482	0.0239	4.81	0.053	Not Significant

## Data Availability

The original contributions presented in this study are included in the article. Further inquiries can be directed to the corresponding authors.
